# Smoking-Related Risks of Colorectal Cancer by Anatomical Subsite and Sex

**DOI:** 10.1093/aje/kwaa005

**Published:** 2020-01-23

**Authors:** Inger T Gram, Song-Yi Park, Lynne R Wilkens, Christopher A Haiman, Loïc Le Marchand

**Affiliations:** 1 Department of Community Medicine, Faculty of Health Sciences, UiT–The Arctic University of Norway, Tromsø, Norway; 2 Population Sciences in the Pacific Program, University of Hawaii Cancer Center, Honolulu, Hawaii; 3 Department of Preventive Medicine, Keck School of Medicine, University of Southern California, Los Angeles, California

**Keywords:** cohort studies, colon cancer, colorectal cancer, multiethnic populations, rectal cancer, sex, smoking

## Abstract

The purpose of this study was to examine whether the increased risk of colorectal cancer due to cigarette smoking differed by anatomical subsite or sex. We analyzed data from 188,052 participants aged 45–75 years (45% men) who were enrolled in the Multiethnic Cohort Study in 1993–1996. During a mean follow-up period of 16.7 years, we identified 4,879 incident cases of invasive colorectal adenocarcinoma. In multivariate Cox regression models, as compared with never smokers of the same sex, male ever smokers had a 39% higher risk (hazard ratio (HR) = 1.39, 95% confidence interval (CI): 1.16, 1.67) of cancer of the left (distal or descending) colon but not of the right (proximal or ascending) colon (HR = 1.03, 95% CI: 0.89, 1.18), while female ever smokers had a 20% higher risk (HR = 1.20, 95% CI: 1.06, 1.36) of cancer of the right colon but not of the left colon (HR = 0.96, 95% CI: 0.80, 1.15). Compared with male smokers, female smokers had a greater increase in risk of rectal cancer with number of pack-years of smoking (*P* for heterogeneity = 0.03). Our results suggest that male smokers are at increased risk of left colon cancer and female smokers are at increased risk of right colon cancer. Our study also suggests that females who smoke may have a higher risk of rectal cancer due to smoking than their male counterparts.

## Abbreviations

CI: confidence interval
CRC: colorectal cancer
HR: hazard ratio
MEC: Multiethnic Cohort
MHT: menopausal hormone therapy

Colorectal cancer (CRC) is the third most common cancer among men and women in the United States. There is substantial variation in tumor anatomical location by sex and age. Women (vs. men) and older (vs. younger) persons have higher risks of developing cancer of the right (proximal or ascending) colon (1, 2). Several authors have advocated that CRC no longer be regarded as a single entity because its anatomical subsites differ with regard to risk factors, incidence, genetic and epigenetic alterations, and prognosis (2–7).

Smoking was established as a causal factor for CRC almost 10 years ago (8, 9). The majority of the studies reviewed considered CRC as a single disease and did not examine possible sex differences. Neither of the 2 expert reports commented on possible sex differences in the relationship between smoking and the 3 anatomical subsites (right (proximal or ascending) colon, left (distal or descending) colon, and rectum) of CRC (8, 9).

In the United States, the prevalence of smoking during the 20th century differed for men and women. Men born between 1911 and 1930 had a smoking prevalence that peaked well above 60%; prevalence in the 1931–1940 birth cohort peaked at around 60%; and the 1941–1950 birth cohort was the last to reach a prevalence above 50%. For females, the 1921–1930 and 1931–1940 birth cohorts were the only two that reached a smoking prevalence above 40%. In 2000, the prevalence of smoking was 25.7% for men and 21.0% for women (10).

In 1996, Giovannucci et al. (11) hypothesized that smoking is an initiator of colorectal carcinogenesis and that tumors emerge 30–40 years after smoking initiation. In the last century, the smoking epidemic in women lagged 10–20 years behind that in men (9, 10, 12), and consequently the smoking-related increase in CRC for women should have been expected to emerge 1 or 2 decades later than that in men.

The purpose of this study was to examine whether the increased risk of CRC due to cigarette smoking differed by tumor anatomical subsite or sex in the Multiethnic Cohort (MEC) Study.

## METHODS

### Study sample

The MEC consists of more than 215,000 men and women who were aged 45–75 years and living in Los Angeles County, California, and the state of Hawaii at the time of cohort recruitment. It comprises mainly 5 racial/ethnic populations (African Americans, Japanese Americans, Latinos, Native Hawaiians, and whites). Briefly, between 1993 and 1996, participants born between 1918 and 1948 enrolled in the study by completing a 26-page mailed questionnaire asking for detailed information on demographic factors, dietary habits, other lifestyle factors, prior medical conditions, and family history of common cancers. We identified potential participants through driver’s license files from state departments of motor vehicles, voter registration lists, and Health Care Financing Administration (Medicare) data files. The institutional review boards of the University of Hawaii and the University of Southern California approved the study protocol. The cohort has been previously described in detail (10).

Of the more than 215,000 MEC Study participants, those who did not belong to one of the 5 main racial/ethnic groups (*n* = 13,987), had prior CRC reported on the baseline questionnaire (*n* = 2,251) or in tumor registries (*n* = 301), had invalid diet information (*n* = 8,116), or had missing information on smoking status (*n* = 2,933) were excluded; this left 188,052 participants (54.8% women) and 4,879 cases with a mean follow-up duration of 16.7 (standard deviation, 5.2) years in the analytical cohort. The multivariable analysis used a complete-case approach, which further excluded subjects with missing data on any of the covariates (*n* = 16,426 including 449 cases), leaving 171,626 participants (53.5% women) and 4,430 CRC cases for these analyses.

### Exposure information

At baseline, participants reported whether they had ever smoked at least 20 packs of cigarettes in their lifetime, the number of years they had smoked cigarettes, the average number of cigarettes smoked per day during the period when they smoked, and, if they had quit smoking, the number of years since they had quit. We calculated pack-years as number of cigarettes smoked per day divided by 20 and multiplied by the duration of smoking in years.

The baseline questionnaire also asked about years of education, height and current weight (for calculation of body mass index (weight (kg)/height (m)^2^)), physical activity (numbers of hours per day spent in moderate and heavy work or recreational activities), and, for women, age at menopause, type of menopause, and ever use of menopausal hormone therapy (MHT). Dietary intake during the previous year was assessed at baseline using a self-administered quantitative food frequency questionnaire with over 180 food items, including alcoholic beverages (13). We calculated mean alcohol intake in grams per day based on the alcohol content of different beverages and usual portion sizes. We calculated daily intakes of nutrients from the questionnaire using a food composition table that was developed and is maintained by the University of Hawaii Cancer Center for the MEC Study.

### Follow-up and endpoints

We identified incident invasive adenocarcinoma of the colon and rectum through linkage with Surveillance, Epidemiology, and End Results Program cancer registries covering Hawaii and California. We classified CRC cases according to anatomical subsite using *International Classification of Diseases for Oncology, Third Edition*, codes: C18.0–C18.5 for the right colon, C18.6–C18.7 for the left colon, and C19.9 and C20.9 for the rectum. We identified deaths through linkage with death certificate files in Hawaii and California and with the National Death Index. Ascertainment of CRC cases and deaths was complete through December 31, 2013. We calculated person-years from cohort entry to the date of CRC diagnosis, death, or the end of follow-up (December 31, 2013), whichever occurred first. CRC cases other than adenocarcinoma (*n* = 324) were censored at the date of diagnosis. Cases with tumors at more than 1 subsite were not included in subsite-specific analyses.

### Statistical analysis

We calculated sex-specific CRC incidence rates per 100,000 person-years, truncated to ages 45–85 years and age-adjusted to the 2000 US standard population (14). Subsequently, we calculated corresponding incidence rates for the 3 anatomical subsites. For each sex, we used Cox proportional hazards regression to model time to CRC, with age as the underlying time scale. We computed hazard ratios and 95% confidence intervals for the associations of CRC with different measures of smoking exposure (smoking status at cohort entry (never, former, current, or ever smoker) and, among ever smokers, smoking duration (≤20, 21–30, or ≥31 years), number of cigarettes smoked per day (≤10, 11–20, or ≥21), and number of pack-years (≤10, 11–20, or ≥21)), using never smokers as the reference group. We repeated the analyses for colon cancer overall and for the 3 subsites.

In the multivariate analyses, we included the following covariates: race/ethnicity (African American, Native Hawaiian, Japanese, Latino, or white; adjusted for as a strata variable), age at cohort entry (years; continuous), family history of CRC (yes, no), history of colorectal polyps (yes, no), body mass index (<25, 25–29.9, or ≥30), multivitamin use (at least once a week during the previous year; yes/no), nonsteroidal antiinflammatory drug use (≥2 times per week for ≥1 month; yes/no), physical activity (hours spent in vigorous work or sports per day), and MHT (never, past, or current use of estrogen) for women.

In addition, we also adjusted for the following dietary intakes: total energy (log-transformed kcal/day), alcohol consumption (g/day), red meat (g/1,000 kcal/day), fiber (g/1,000 kcal/day), calcium (mg/day), folate (dietary folate equivalents per day), and vitamin D (IU/day). We modeled the dietary intake variables in the multivariate Cox models as continuous variables.

Participants with missing data on covariates tended to be older (63.7 years vs. 59.5 years), female (69.1% vs. 53.5%), and never smokers (47.4% vs. 43.7%) and to have similar proportions of CRC cases (2.7% vs. 2.6%) compared with those with complete data. We present the results from the complete-case analyses throughout this paper. The proportional hazards assumption was tested using Schoenfeld residuals and was found to hold (15, 16).

We conducted tests for linear trends by including an ordinal exposure variable with equally spaced scores in the models, using never smokers as the first category. We assessed heterogeneity in the CRC-smoking association by sex on the basis of Wald statistics for cross-product terms between sex and smoking trend variables. We calculated CRC-smoking associations with adjustment for MHT (which was set to 0 in men) and other covariates using sex as a covariate. The effect of the interaction between smoking and MHT in women on the risk of CRC was similarly tested. We performed the analyses using SAS, version 9.4 (SAS Institute Inc., Cary, North Carolina).

## RESULTS

For men and women, the annual age-adjusted incidence rates of CRC were 126.4 per 100,000 person-years and 91.8 per 100,000 person-years (truncated to ages 45–85 years), respectively. For men, the distribution of cases by anatomical subsite was 42.6% right colon, 29.2% left colon, 26.2% rectum, and 2.0% unknown. The corresponding numbers for women were 52.8%, 26.3%, 18.3% and 2.6%, respectively. At enrollment, 18.2% of men and 14.4% of women were current smokers. Altogether, 70% of men and 44% of women reported ever smoking.

Table 1 shows that, for both sexes, age at diagnosis was lower for ever smokers than for never smokers. The right colon was the subsite at which cancer was most frequently diagnosed among both ever smokers and never smokers of both sexes. Except for left colon cancer among women, ever smokers had higher sex-specific incidence rates than never smokers for all subsites. Among never smokers, men had higher incidence rates than women for right colon cancer and rectal cancer and a similar incidence rate as women for left colon cancer. Compared with female smokers, male smokers had smoked for more years, smoked more cigarettes per day, and, consequently, had more pack-years of smoking.

**Table 1:** Selected Baseline Characteristics of Men and Women Followed Through 2013, by Smoking Status, Multiethnic Cohort Study, 1993–1996

**Characteristic**	**Men (*n* = 84,948)**						**Women (*n* = 103,104)**								
	**Ever** **Smokers** **(*n* = 59,491)**				**Never Smokers** **(*n* = 25,457)**			**Ever Smokers** **(*n* = 45,724)**			**Never Smokers** **(*n* = 57,380)**			**Total** **(*n* = 188,052)**		
	**Mean** **(SD)**	**%**	**IR^a^**	**Mean** **(SD)**	**%**	**IR**	**Mean** **(SD)**	**%**	**IR**	**Mean (SD)**	**%**	**IR**	**Mean** **(SD)**	**%**	**IR**
Age at cohort entry, years	60.5 (8.7)			59.4 (9.1)			59.0 (8.7)			60.2 (8.9)			59.9 (8.9)		
Age at diagnosis, years^b^	72.3 (8.5)			72.8 (8.9)			72.2 (8.9)			74.0 (9.2)			72.8 (8.9)		
CRC subtype^c^															
Colorectum			138.4			101.5			101.5			84.8			106.9
Colon			97.4			74.1			78.9			68.9			80.4
Right colon			53.5			46.2			50.8			42.1			48.0
Left colon			41.1			25.2			24.9			25.5			30.0
Rectum			39.8			26.4			21.8			15.3			25.7
Duration of smoking, years	23.3 (12.5)						21.2 (12.5)						22.4 (12.6)		
No. of cigarettes smoked per day	16.1 (8.5)						12.7 (7.6)						14.7 (8.3)		
Pack-years of smoking	20.6 (16.6)						15.4 (14.4)						18.4 (15.9)		
Family history of colorectal cancer		7.2			7.3			8.6			8.6			8.0	
History of intestinal polyps		7.3			5.9			4.6			4.2			5.5	
Body mass index^d^															
<25		35.6			37.2			43.7			48.2			41.6	
25–29.9		46.8			46.6			32.2			31.4			38.6	
≥30		17.6			16.2			24.0			20.4			19.8	
Physical activity, hours/day^e^	0.57 (1.02)			0.62 (1.03)			0.23 (0.59)			0.19 (0.51)			0.38 (0.82)		
Daily dietary intake															
Energy, kcal	2,445 (1,140)			2,375 (1,099)			1,985 (976)			1,970 (953)			2,179 (1,065)		
Alcohol, g	16.9 (35.7)			9.7 (23.9)			6.9 (19.5)			2.3 (9.5)			9.0 (25.2)		
Red meat, g/1,000 kcal	20.5 (13.0)			19.1 (12.7)			17.9 (12.6)			16.5 (11.8)			18.4 (12.6)		
Fiber, g/1,000 kcal	10.5 (4.0)			11.5 (4.1)			12.0 (4.2)			13.0 (4.2)			11.8 (4.2)		
Calcium, mg^f^	1,008 (613)			1,054 (633)			1,081 (729)			1,136 (747)			1,071 (689)		
Folate, μg of DFE^f^	970 (624)			1,022 (647)			903 (603)			937 (604)			951 (617)		
Vitamin D, IU^f^	338 (342)			353 (351)			343 (352)			344 (344)			343 (347)		
Multivitamin use		47.1			48.8			53.3			54.4			51.1	
NSAID use		52.7			46.9			58.8			49.7			52.5	
Menopausal hormone therapy															
Never use								51.4			54.9				
Past use								19.4			16.9				
Current use								29.1			28.3				

Abbreviations: CRC, colorectal cancer; DFE, dietary folate equivalents; IR, incidence rate; NSAID, nonsteroidal antiinflammatory drug; SD, standard deviation.

^a^ Number of cases per 100,000 person-years.

^b^ Among men and women with incident CRC.

^c^ Rates, truncated to ages 45–85 years, were adjusted to the 2000 US standard population.

^d^ Weight (kg)/height (m)^2^.

^e^ Amount of time spent in vigorous work or sports per day.

^f^ From foods and supplements.

Web Table 1 (available at https://academic.oup.com/aje) shows the direct associations of smoking status and the 3 measures of smoking dose/duration (number of cigarettes per day, number of years of smoking, and number of pack-years) with multivariate-adjusted CRC risk overall for each sex (all *P-*for-trend values < 0.001). These associations did not differ by sex (all *P-*for-heterogeneity values > 0.13).

Table 2 shows that the race/ethnicity- and age-adjusted estimates, compared with the multivariable-adjusted estimates, were quite similar for colon cancer overall. For both sexes, we observed direct associations with colon cancer risk overall for the 4 smoking measures (all *P-*for-trend values < 0.05), with no sex differences (all *P-*for-heterogeneity values > 0.43).

**Table 2:** Association Between Smoking and Colon Cancer Risk, by Sex, Multiethnic Cohort Study, 1993–2013

**Smoking Exposure**	**Men (*n* = 84,948)**						**Women (*n* = 103,104)**						***P* for** **Heterogeneity** ^**d**^
	**No. of** **Cases**	**HR** ^**a**^	**95% CI**	**No. of** **Cases** ^**b**^	**HR** ^**c**^	**95% CI**	**No. of** **Cases**	**HR** ^**a**^	**95% CI**	**No. of** **Cases** ^**b**^	**HR** ^**c**^	**95% CI**	
None (never smokers)	489	1.00	Referent	464	1.00	Referent	1,055	1.00	Referent	919	1.00	Referent	
Smoking status													0.43
Former smoker	1,050	1.20	1.08, 1.34	974	1.14	1.02, 1.27	609	1.17	1.06, 1.30	534	1.16	1.04, 1.30	
Current smoker	307	1.28	1.11, 1.48	287	1.20	1.03, 1.39	241	1.15	1.00, 1.33	205	1.07	0.91, 1.25	
Ever smoker	1,357	1.22	1.10, 1.35	1,261	1.15	1.03, 1.28	850	1.17	1.06, 1.28	739	1.14	1.03, 1.26	0.80
Ever smokers													
Duration of smoking, years													0.55
≤20	533	1.16	1.03, 1.32	499	1.12	0.99, 1.28	363	1.07	0.94, 1.20	313	1.05	0.92, 1.19	
21–30	312	1.25	1.08, 1.44	298	1.19	1.03, 1.38	201	1.31	1.12, 1.53	176	1.26	1.07, 1.49	
≥31	476	1.26	1.11, 1.43	434	1.14	1.00, 1.31	257	1.24	1.08, 1.42	230	1.21	1.04, 1.40	
*P* for trend		<0.001			0.04			<0.001			0.002		
No. of cigarettes smoked per day													0.44
≤10	436	1.15	1.01, 1.31	398	1.12	0.98, 1.28	428	1.07	0.95, 1.19	362	1.04	0.92, 1.17	
11–20	482	1.18	1.04, 1.34	446	1.09	0.96, 1.24	280	1.31	1.14, 1.50	248	1.26	1.09, 1.46	
≥21	401	1.35	1.18, 1.54	385	1.25	1.09, 1.43	127	1.35	1.12, 1.64	117	1.31	1.07, 1.60	
*P* for trend		<0.001			0.005			<0.001			<0.001		
Pack-years of smoking													0.48
≤10	374	1.17	1.02, 1.34	345	1.14	0.99, 1.31	331	1.08	0.95, 1.23	284	1.06	0.93, 1.22	
11–20	414	1.15	1.01, 1.31	388	1.09	0.95, 1.25	263	1.16	1.01, 1.33	227	1.11	0.96, 1.29	
≥21	513	1.32	1.16, 1.49	480	1.20	1.05, 1.37	221	1.37	1.18, 1.59	203	1.33	1.14, 1.56	
*P* for trend		<0.001			0.01			<0.001			<0.001		

Abbreviations: CI, confidence interval; HR, hazard ratio.

^a^ Adjusted for race/ethnicity and age at cohort entry.

^b^ Participants with missing information on covariates were excluded.

^c^ Results were further adjusted for family history of colorectal cancer, history of colorectal polyps, body mass index, physical activity, multivitamin use, nonsteroidal antiinflammatory drug use, menopausal hormone therapy (for women only), and intakes of alcohol, total energy, red meat, dietary fiber, calcium, folate, and vitamin D.

^d^ Tests for heterogeneity between men and women were performed on the basis of joint multivariate-adjusted models with adjustment for sex as a strata variable.

Table 3 shows that for men, compared with never smokers, ever smokers had a similar risk of right colon cancer (hazard ratio (HR) = 1.03, 95% confidence interval (CI): 0.89, 1.18) and a 39% higher risk of left colon cancer (HR = 1.39, 95% CI: 1.16, 1.67). We observed direct associations between the different measures of smoking dose/duration and cancer of the left colon (all *P-*for-trend values < 0.001) but not cancer of the right colon (all *P-*for-trend values > 0.66). For women, compared with never smokers, ever smokers had a 20% higher risk of right colon cancer (HR = 1.20, 95% CI: 1.06, 1.36). We observed direct associations between the different measures of smoking exposure and right colon cancer (all *P-*for-trend values < 0.001) but not left colon cancer (all *P-*for-trend values > 0.50). There was a sex difference for all 3 measures of smoking dose/duration displayed (all *P-*for-heterogeneity values ≤ 0.01).

**Table 3:** Associations Between Smoking and Right and Left Colon Cancer Risk, by Sex, Multiethnic Cohort Study, 1993–2013^a^

**Smoking Exposure**	**Right Colon**							**Left Colon**						
	**Men**			**Women**				**Men**			**Women**			
	**No. of** **Cases**	**HR**	**95% CI**	**No. of** **Cases**	**HR**	**95% CI**	***P* for** **Heterogeneity** ^**b**^	**No. of** **Cases**	**HR**	**95% CI**	**No. of** **Cases**	**HR**	**95% CI**	***P* for** **Heterogeneity** ^**b**^
None (never smokers)	294	1.00	Referent	581	1.00	Referent		155	1.00	Referent	318	1.00	Referent	
Smoking status							0.43							0.01
Former smoker	547	1.01	0.87, 1.16	354	1.21	1.06, 1.39		410	1.42	1.18, 1.71	156	1.01	0.83, 1.23	
Current smoker	159	1.10	0.90, 1.35	135	1.15	0.95, 1.41		115	1.31	1.02, 1.68	62	0.86	0.64, 1.14	
Ever smoker	706	1.03	0.89, 1.18	489	1.20	1.06, 1.36	0.14	525	1.39	1.16, 1.67	218	0.96	0.80, 1.15	0.004
Ever smokers														
Duration of smoking, years							0.01							0.007
≤20	294	1.06	0.90, 1.25	203	1.07	0.91, 1.26		197	1.30	1.05, 1.61	97	0.93	0.74, 1.18	
21–30	167	1.08	0.89, 1.30	113	1.31	1.06, 1.61		123	1.42	1.12, 1.80	59	1.16	0.87, 1.55	
≥31	228	0.95	0.80, 1.14	159	1.32	1.10, 1.59		192	1.48	1.19, 1.84	57	0.87	0.65, 1.17	
*P* for trend		0.67			<0.001				<0.001			0.68		
No. of cigarettes smoked per day							0.007							0.006
≤10	240	1.05	0.88, 1.24	230	1.04	0.89, 1.21		146	1.25	1.00, 1.58	117	0.98	0.79, 1.22	
11–20	237	0.93	0.78, 1.11	172	1.41	1.18, 1.68		199	1.40	1.14, 1.74	63	0.89	0.68, 1.18	
≥21	205	1.10	0.91, 1.32	80	1.47	1.15, 1.87		172	1.56	1.24, 1.95	34	1.04	0.72, 1.50	
*P* for trend		0.67			<0.001				<0.001			0.73		
Pack-years of smoking							0.005							0.003
≤10	215	1.12	0.93, 1.33	184	1.08	0.92, 1.28		124	1.22	0.96, 1.55	87	0.95	0.75, 1.21	
11–20	197	0.88	0.74, 1.06	142	1.12	0.93, 1.35		177	1.46	1.18, 1.82	79	1.09	0.84, 1.40	
≥21	261	1.06	0.89, 1.26	147	1.56	1.29, 1.88		209	1.48	1.19, 1.83	44	0.81	0.58, 1.12	
*P* for trend		0.96			<0.001				<0.001			0.51		

Abbreviations: CI, confidence interval; HR, hazard ratio.

^a^ Adjusted for race/ethnicity, age at cohort entry, family history of colorectal cancer, history of colorectal polyps, body mass index, physical activity, multivitamin use, nonsteroidal antiinflammatory drug use, menopausal hormone therapy (for women only), and intakes of alcohol, total energy, red meat, dietary fiber, calcium, folate, and vitamin D.

^b^ Tests for heterogeneity between men and women were performed on the basis of joint multivariate-adjusted models with adjustment for sex as a strata variable.

Web Table 2 shows that among postmenopausal women, both ever and never users of MHT had statistically significantly higher smoking-related multivariate-adjusted risks of right colon cancer. Among MHT ever users, several of the corresponding risk estimates for left colon cancer were above unity but not statistically significant. The test for heterogeneity across MHT status for the association between smoking status (former, current, or ever smoker) and left colon cancer risk was statistically significant (both *P*-for-heterogeneity values = 0.02).

Table 4 shows that ever smokers, compared with never smokers, had higher risks of rectal cancer: 40% higher (HR = 1.40, 95% CI: 1.16, 1.69) for men and 58% higher (HR = 1.58, 95% CI: 1.28, 1.95) for women. For both sexes, we observed direct associations between the 3 measures of smoking dose/duration and rectal cancer risk (all *P-*for-trend values < 0.001). We found a greater increase in risk of rectal cancer with number of pack-years for women than for men (*P* for heterogeneity = 0.03).

**Table 4:** Association Between Smoking and Rectal Cancer Risk, by Sex, Multiethnic Cohort Study, 1993–2013^a^

**Smoking Exposure**	**Men**			**Women**			***P* for** **Heterogeneity** ^**b**^
	**No. of** **Cases**	**HR**	**95% CI**	**No. of** **Cases**	**HR**	**95% CI**	
None (never smokers)	147	1.00	Referent	184	1.00	Referent	
Smoking status							0.78
Former smoker	327	1.26	1.04, 1.54	132	1.49	1.19, 1.88	
Current smoker	153	1.90	1.50, 2.40	69	1.80	1.34, 2.42	
Ever smoker	480	1.40	1.16, 1.69	201	1.58	1.28, 1.95	0.33
Ever smokers							
Duration of smoking, years							0.17
≤20	177	1.26	1.01, 1.56	73	1.24	0.94, 1.64	
21–30	105	1.33	1.03, 1.72	53	1.95	1.42, 2.67	
≥31	190	1.68	1.35, 2.11	70	1.90	1.42, 2.55	
*P* for trend		<0.001			<0.001		
No. of cigarettes smoked per day							0.10
≤10	151	1.40	1.11, 1.76	98	1.46	1.13, 1.87	
11–20	167	1.30	1.04, 1.62	65	1.65	1.23, 2.21	
≥21	150	1.53	1.21, 1.94	33	1.77	1.20, 2.60	
*P* for trend		<0.001			<0.001		
Pack-years of smoking							0.03
≤10	140	1.47	1.16, 1.85	66	1.27	0.95, 1.69	
11–20	125	1.12	0.88, 1.43	65	1.65	1.24, 2.21	
≥21	198	1.61	1.29, 2.00	63	2.09	1.54, 2.83	
*P* for trend		<0.001			<0.001		

Abbreviations: CI, confidence interval; HR, hazard ratio.

^a^ Adjusted for race/ethnicity, age at cohort entry, family history of colorectal cancer, history of colorectal polyps, body mass index, physical activity, multivitamin use, nonsteroidal antiinflammatory drug use, menopausal hormone therapy (for women only), and intakes of alcohol, total energy, red meat, dietary fiber, calcium, folate, and vitamin D.

^b^ Tests for heterogeneity between men and women were performed on the basis of joint multivariate-adjusted models with adjustment for sex as a strata variable.

**Figure 1:**
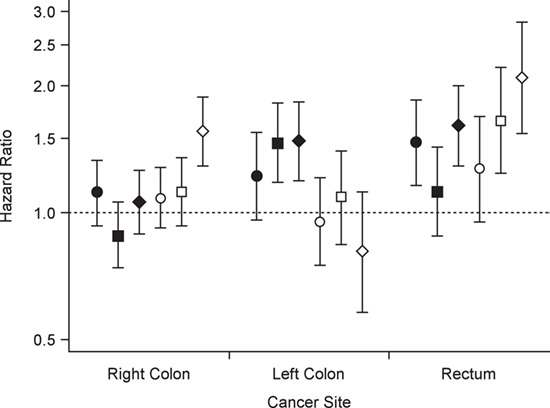
Multivariate-adjusted risks of right and left colon cancer and rectal cancer according to pack-years of smoking, by sex, Multiethnic Cohort Study, 1993–2013. Results were adjusted for race/ethnicity, age at cohort entry, family history of colorectal cancer, history of colorectal polyps, body mass index, physical activity, multivitamin use, nonsteroidal antiinflammatory drug use, menopausal hormone therapy (for women only), and intakes of alcohol, total energy, red meat, dietary fiber, calcium, folate, and vitamin D. The reference group was never smokers. Circles, ≤10 pack-years of smoking; squares, 11–20 pack-years; diamonds, ≥21 pack-years. Black markers show results for men and white markers results for women. Bars, 95% confidence intervals.

Figure 1 displays multivariate-adjusted risks of cancer at the 3 anatomical subsites according to pack-years of smoking, by sex. The figure shows that the association differs by sex for left and right colon cancer and that the association between smoking and rectal cancer is stronger for women than for men.

## DISCUSSION

In the present prospective study, although women were smoking less than men, we found that the smoking-related increased risks for CRC and for colon cancer overall were similar for both sexes. However, we observed that this increase in colon cancer risk for men was confined to the left colon, and for women to the right colon. Additionally, among postmenopausal women, for both ever and never users of MHT, we found a smoking-related increase in the risk of right colon cancer. Furthermore, while the association between smoking and rectal cancer was present for both men and women, it was stronger for women.

We had previously examined the sex-specific associations between smoking and colon (17) and rectal (18) cancer in a large Norwegian cohort including 600,000 men and women. The results from these studies suggested that female smokers may be more susceptible to colon cancer (17) but not rectal cancer (18) in comparison with male smokers. Male former smokers had a higher risk of left colon cancer (17). In the MEC, we observed that both current and former male smokers had a higher risk of left colon cancer.

As we pointed out in the Introduction, women in the United States took up smoking in large numbers more recently than men (9, 10, 12). The present study included the birth cohorts of men and women that have had the highest smoking prevalences in US history. In the MEC, we find that men have a higher incidence rate of CRC, smoke more, and have a smaller proportion of never smokers compared with women. These findings are all in accordance with population reports from both the United States (10, 19) and Norway (20, 21).

Differences in incidence patterns for lung cancer, a cancer driven largely by smoking behavior, between the United States and Norway provides insight into the distinct results observed by country. As a result of reductions in smoking prevalence that began decades earlier (9, 10, 12), lung cancer incidence rates began declining in the United States in the mid-1980s in men and in the mid-2000s in women (19). In Norway, not until 2013 did the lung cancer incidence among men start to decline, while it was still increasing among women (20). We argue that the 2 main reasons for the stronger and more consistent sex differences for CRC risk in the MEC as compared with the Norwegian cohort are 1) the lag time in the decline in the smoking epidemic in Norway compared with the United States and 2) the fact that follow-up in the Norwegian cohort extended only to 2007, as compared with 2013 in the MEC.

In further support of this notion are the results from one meta-analysis including older cohorts (22) and those of another including more recent cohorts (5). The former meta-analysis found that, compared with sex-specific never smokers, male smokers had a nearly 40% statistically significantly higher risk of CRC, while female smokers had a 6% higher risk of CRC that was not statistically significant (22). In the latter study, the smoking-related increases in risk for both overall colon cancer and rectal cancer were quite similar for male and female smokers.

The remaining cohort studies included only women (23–27) or did not report results by sex (28, 29). In the most recent report from the European Prospective Investigation into Cancer and Nutrition, with more than 1,700 left colon cancer cases, Murphy et al. (29) found a positive relationship between smoking and left colon cancer for former smokers but not for current smokers. Since the report did not stratify results by sex, we can only speculate that the relationship was predominately seen in men, since more men (35%) than women (23%) were former smokers.

Smoking cigarettes causes exposure to a mixture of more than 7,000 toxic chemicals, including at least 70 known carcinogens that can affect nearly every organ system in the human body (30). Carcinogens in cigarette smoke, such as nitrosamines, heterocyclic amines, benzene, and polycyclic aromatic hydrocarbons, may reach the colorectal mucosa through direct ingestion or through the bloodstream and may have a direct oncogenic effect on both the colon and the rectum (5). The evidence suggests that smoking probably plays a role in early carcinogenesis in both the colon and the rectum, as reflected by its association with colorectal adenomas. The temporal pattern of the effects of smoking, with a continuing increase in risk, particularly for rectal cancer, suggests that smoking may also act in the later stages of CRC carcinogenesis (9).

Several reports have described biological mechanisms by which smoking may cause CRC (5, 6, 8, 9, 28, 31–36). In the Iowa Women’s Health Study, smoking was associated with the microsatellite-instability–high, CpG island methylator phenotype–positive, and B-Raf protein encoding gene (*BRAF*) mutation–positive subtypes of CRC, which indicates that epigenetic modification may be functionally involved in smoking-related colorectal carcinogenesis (33). These results were later confirmed in a meta-analysis examining correlations between smoking history and molecular pathways in sporadic CRC (6). Differences in genetic makeup and lifestyle, including smoking and dietary habits, are thought to cause the differences between right- and left-sided colon cancer (2). Our findings that smoking increases the risks of right- and left-sided colon cancer among women and men, respectively, fit with the overall sex-specific site predominance for colon cancer.

In the United States, males have had a higher incidence of lung cancer than of CRC for many decades, while females had higher CRC incidence than lung cancer incidence until the early 1990s. For males, the decrease in CRC incidence also started in the early 1990s and paralleled that of lung cancer incidence. For females, CRC incidence rates have also been decreasing steadily since the mid-1980s, while the decrease for lung cancer started later (19).

CRC incidence rates for people aged ≥50 years peaked in 1985 in the United States (37). The changes in CRC incidence result from changes in risk factor prevalences and levels and CRC screening practices (38). Right colon cancer is associated with more aggressive disease and has a worse prognosis than left colon cancer (4). In our study, the right colon was the most frequent anatomical subsite of CRC. Right colon cancer was diagnosed in a larger proportion of participants for both sexes in our study, as compared with the sex-specific proportion of right colon cancer reported for the US population (1).

Our study had several major strengths. We had a high proportion of ever smokers in both sexes. We obtained smoking histories at enrollment and, thus, they were unlikely to have been subject to recall bias. In the United States, hardly anyone starts to smoke after age 50 years, which is similar to our minimum enrollment age of 45 years (9). Furthermore, we have previously demonstrated the internal validity of the smoking exposure variables (39–41) and associations with other lifestyle factors (dietary fiber intake, MHT, and alcohol consumption) and the CRC outcome (42, 43). We had detailed information on, and were able to control for, the established risk factors for CRC, many of which vary according to smoking status.

The main limitation of our study is that despite having close to 5,000 incident CRC cases, the numbers of cases were relatively small for some subset analyses. The lack of statistical significance for the association with left colon cancer among ever users of MHT may have been a function of low statistical power rather than the strength of the association. We plan to examine whether there are similar sex differences regarding tumor location in each of the 5 racial/ethnic groups when we have a longer duration of follow-up. The latency interval between smoking initiation and CRC diagnosis is presumed to be several decades (44). In our study, there were more ever smokers among men than among women. Therefore, more men than women will have died from other smoking-related diseases during follow-up, before they could be diagnosed with CRC. This may have deflated the risk estimates more in men than in women. Furthermore, the results from a recent meta-analysis suggest that passive smoking is associated with an increased risk of CRC, especially of rectal cancer (45). We lacked information about passive smoking. This may have influenced our results differently according to sex, since more women than men are never smokers. Thus, our CRC risk estimates could have been attenuated more for women than for men, since never-smoking participants who were passively exposed to smoking were included in the sex-specific reference groups.

We need more knowledge about the biology of the sex differences for CRC in general and for the smoking-related differences specifically. We strongly recommend that cohort studies examining risk factors for CRC do this by sex and subsite. In addition, animal studies need to include females, which is rarely done today (2). Increased understanding of sex differences in CRC development could, in the future, lead to sex-specific counseling for CRC prevention, as well as different CRC screening strategies and more personalized treatment for men and women.

The smoking-attributable proportion of CRC cases has started to emerge for women during the last decade and could, at least temporarily, be greater in women than men in some populations. We hypothesize that our study will be the first of many cohort studies demonstrating that the increased risks of both colon and rectal cancer due to cigarette smoking are greater in women than in men.

In conclusion, our results suggest that women who smoke have an increased risk of right colon cancer, whereas men who smoke have an increased risk of left colon cancer. Women may also have a higher risk of rectal cancer due to smoking than men.

## Supplementary Material

kwaa005_Gram_Web_Material_Final
