# Supplementary material for: Smoking-Related Risks of Colorectal Cancer by Anatomical Subsite and Sex
Source: Am J Epidemiol. 2020 Jan 23;189(6):543–53. doi: 10.1093/aje/kwaa005 (PMC7368133; doi:10.1093/aje/kwaa005)
Supplement: kwaa005_Gram_Web_Material_Final [file kwaa005_gram_web_material_final.pdf]

Smoking-Related Risks of Colorectal Cancer by Anatomical Subsite and Sex

Inger T. Gram[1], Song-Yi Park[2], Lynne R. Wilkens[2], Christopher A. Haiman[3], and Loïc Le Marchand[2]

Table of Contents:

- Web Table 1
- Web Table 2

Web Table 1. Smoking and colorectal cancer risk by sex in the Multiethnic Cohort Study, 1993–2013

Smoking Exposure	Men (*n* = 84,948)				Women (*n* = 103,104)				$P_{heterogeneity}$[d]
	Cases	HR (95% CI)[a]	Cases[b]	HR (95% CI)[c]	Cases	HR (95% CI)[a]	Cases[b]	HR (95% CI)[c]	
Common reference group									
Never smokers	648	1.00 (ref)	617	1.00 (ref)	1264	1.00 (ref)	1109	1.00 (ref)	
Smoking status									0.27
Former	1414	1.23 (1.12-1.35)	1314	1.17 (1.06-1.28)	767	1.24 (1.13-1.36)	671	1.22 (1.11-1.35)	
Current	469	1.47 (1.31-1.66)	442	1.36 (1.20-1.55)	317	1.26 (1.11-1.43)	277	1.19 (1.04-1.37)	
Ever	1883	1.28 (1.17-1.40)	1756	1.21 (1.10-1.32)	1084	1.25 (1.14-1.35)	948	1.21 (1.11-1.33)	0.96
Ever smokers									
Smoking duration, y									0.33
≤20	724	1.19 (1.07-1.32)	681	1.15 (1.03-1.29)	450	1.10 (0.99-1.23)	389	1.08 (0.96-1.22)	
21-30	427	1.29 (1.14-1.45)	407	1.23 (1.08-1.39)	258	1.40 (1.22-1.61)	229	1.37 (1.18-1.58)	
≥31	685	1.40 (1.26-1.56)	630	1.27 (1.13-1.42)	342	1.38 (1.22-1.56)	305	1.34 (1.17-1.52)	
P_{trend}		<0.001		<0.001		<0.001		<0.001	
No. of cigarettes									0.19
≤10/day	603	1.22 (1.09-1.37)	554	1.18 (1.05-1.33)	543	1.14 (1.03-1.26)	464	1.11 (0.99-1.24)	
11-20/day	661	1.22 (1.09-1.36)	617	1.14 (1.01-1.27)	354	1.38 (1.22-1.55)	315	1.32 (1.16-1.51)	
≥21/day	567	1.43 (1.28-1.60)	541	1.32 (1.17-1.48)	166	1.46 (1.23-1.72)	152	1.40 (1.17-1.67)	
P_{trend}		<0.001		<0.001		<0.001		<0.001	
Pack-years									0.14
≤10	524	1.24 (1.11-1.39)	489	1.21 (1.08-1.37)	409	1.12 (1.00-1.25)	353	1.10 (0.98-1.24)	
11-20	551	1.16 (1.03-1.30)	518	1.10 (0.98-1.24)	336	1.24 (1.10-1.41)	294	1.21 (1.06-1.38)	
≥21	732	1.43 (1.28-1.59)	684	1.29 (1.16-1.45)	296	1.53 (1.34-1.74)	269	1.47 (1.27-1.69)	
P_{trend}		<0.001		<0.001		<0.001		<0.001	

[a]Adjusted for race/ethnicity and age at cohort entry.
[b]Excluding participants with missing information on covariates.
[c]Further adjusted for family history of colorectal cancer, history of colorectal polyp, body mass index, multivitamin use, non-steroidal anti-inflammatory drug use, menopausal hormone therapy use for women only , and intake of alcohol, total energy, red meat, dietary fiber, calcium, folate, and vitamin D.
[d]Tests for heterogeneity between men and women were performed based on the multivariate-adjusted models.

Web Table 2. Smoking and right and left colon cancer risk among postmenopausal women by MHT use in the Multiethnic Cohort Study, 1993–2013

Smoking Exposure	Right Colon					Left Colon				
	MHT never users		MHT ever users		$P_{heterogeneity}$[b]	MHT never users		MHT ever users		$P_{heterogeneity}$[b]
	Cases	HR (95% CI)[a]	Cases	HR (95% CI)[a]		Cases	HR (95% CI)[a]	Cases	HR (95% CI)[a]	
Common reference group										
Never smokers	269	1.00 (ref)	260	1.00 (ref)		161	1.00 (ref)	114	1.00 (ref)	
Smoking status					0.68					0.02
Former	161	1.34 (1.10-1.64)	159	1.10 (0.90-1.35)		60	0.87 (0.64-1.18)	78	1.21 (0.90-1.63)	
Current	59	1.12 (0.84-1.51)	68	1.47 (1.11-1.94)		25	0.69 (0.44-1.07)	28	1.26 (0.82-1.95)	
Ever	220	1.28 (1.06-1.54)	227	1.18 (0.98-1.43)	0.52	85	0.81 (0.62-1.07)	106	1.22 (0.93-1.62)	0.02
Ever smokers										
Smoking duration, y					0.74					0.16
≤20	87	1.17 (0.92-1.50)	90	1.00 (0.78-1.28)		33	0.75 (0.52-1.10)	51	1.29 (0.92-1.80)	
21-30	47	1.40 (1.02-1.92)	52	1.30 (0.96-1.77)		18	0.82 (0.50-1.35)	28	1.50 (0.98-2.29)	
≥31	78	1.38 (1.06-1.80)	79	1.38 (1.06-1.79)		31	0.88 (0.59-1.31)	26	0.99 (0.64-1.55)	
P_{trend}		0.005		0.01			0.32		0.44	
No. of cigarettes					0.13					0.08
≤10/day	96	1.01 (0.80-1.28)	111	1.07 (0.85-1.35)		49	0.90 (0.65-1.24)	52	1.15 (0.82-1.61)	
11-20/day	84	1.69 (1.31-2.18)	79	1.36 (1.04-1.76)		18	0.54 (0.33-0.89)	37	1.39 (0.95-2.04)	
≥21/day	36	1.71 (1.19-2.45)	34	1.26 (0.87-1.83)		17	1.16 (0.69-1.96)	15	1.15 (0.66-2.01)	
P_{trend}		<0.001		0.03			0.25		0.17	
Pack-years					0.17					0.08
≤10	75	1.10 (0.85-1.43)	89	1.10 (0.86-1.41)		31	0.79 (0.53-1.16)	45	1.28 (0.90-1.82)	
11-20	58	1.11 (0.83-1.49)	69	1.18 (0.90-1.55)		31	0.93 (0.63-1.37)	37	1.38 (0.94-2.02)	
≥21	77	1.89 (1.44-2.47)	63	1.35 (1.01-1.80)		19	0.68 (0.42-1.12)	22	1.00 (0.62-1.62)	
P_{trend}		<0.001		0.03			0.14		0.37	

Abbreviation: MHT, menopausal hormone therapy.

[b]Adjusted for race/ethnicity, age at cohort entry, family history of colorectal cancer, history of colorectal polyp, body mass index, physical activity, multivitamin use, non-steroidal anti-inflammatory drug use, and intake of alcohol, total energy, red meat, dietary fiber, calcium, folate, and vitamin D.

[b]Tests for heterogeneity between men and women were performed based on the multivariate-adjusted models.
